# Coordinated spatial and temporal expression of *Hox *genes during embryogenesis in the acoel *Convolutriloba longifissura*

**DOI:** 10.1186/1741-7007-7-65

**Published:** 2009-10-01

**Authors:** Andreas Hejnol, Mark Q Martindale

**Affiliations:** 1 Kewalo Marine Laboratory, PBRC, University of Hawaii, 41 Ahui Street, Honolulu, HI 96813, USA; 2 Sars International Centre for Marine Molecular Biology, University of Bergen, Thormøhlensgaten 55, 5008 Bergen, Norway

## Abstract

**Background:**

*Hox *genes are critical for patterning the bilaterian anterior-posterior axis. The evolution of their clustered genomic arrangement and ancestral function has been debated since their discovery. As acoels appear to represent the sister group to the remaining Bilateria (Nephrozoa), investigating *Hox *gene expression will provide an insight into the ancestral features of the *Hox *genes in metazoan evolution.

**Results:**

We describe the expression of anterior, central and posterior class *Hox *genes and the *ParaHox *ortholog *Cdx *in the acoel *Convolutriloba longifissura*. Expression of all three *Hox *genes begins contemporaneously after gastrulation and then resolves into staggered domains along the anterior-posterior axis, suggesting that the spatial coordination of *Hox *gene expression was present in the bilaterian ancestor. After early surface ectodermal expression, the anterior and central class genes are expressed in small domains of putative neural precursor cells co-expressing *ClSoxB1*, suggesting an evolutionary early function of *Hox *genes in patterning parts of the nervous system. In contrast, the expression of the posterior *Hox *gene is found in all three germ layers in a much broader posterior region of the embryo.

**Conclusion:**

Our results suggest that the ancestral set of *Hox *genes was involved in the anterior-posterior patterning of the nervous system of the last common bilaterian ancestor and were later co-opted for patterning in diverse tissues in the bilaterian radiation. The lack of temporal colinearity of *Hox *expression in acoels may be due to a loss of genomic clustering in this clade or, alternatively, temporal colinearity may have arisen in conjunction with the expansion of the *Hox *cluster in the Nephrozoa.

## Background

*Hox *genes encode transcription factors that contain a characteristic helix-turn-helix DNA binding domain - the homeodomain. *Hox *genes regulate the expression of other genes during development and their molecular characterization and expression patterns indicate that these genes are involved in specifying regional identities along the anterior posterior (A-P) axis in a diverse range of bilaterian animals [1,2]. Orthologs of the *Hox *genes are subdivided into anterior, central and posterior classes, according to their suspected evolutionary ancestry and their corresponding expression along the A-P axis. Surprisingly, in most, but not all, bilaterians whose genomes have been sequenced, the *Hox *genes are organized in a contiguous cluster in which the order of genes along the chromosome is reflected in their expression domains along the A-P axis, a phenomenon called 'spatial colinearity' [3-7]. Additionally, in some cases the developmental timing of expression of sequential *Hox *genes also corresponds to their relative positions in the genome - a phenomenon that has been described as 'temporal colinearity' [8-10]. Both patterns suggest that spatial and temporal colinearity might have been present in the organism that possessed the ancestral *Hox *cluster [11].

Similar attention has been given to the *ParaHox *genes, a group of genes that are also clustered in some animal genomes (for example, *Branchiostoma*) and are thought to be paralogs of the *Hox *genes [12]. Phylogenetic evidence from bilaterian taxa suggests that each of the three known *ParaHox *gene classes (*Gsx*, *Xlox *and *Cdx*) is more closely related to a corresponding *Hox *class than to each other. This suggests that the *Hox *cluster and *ParaHox *cluster arose by the duplication of a single proto-Hox cluster prior to the separation of the cnidarian and bilaterian lineages [12-14]. Organized clusters of *Hox *genes, composed of anterior class (paralog groups *Hox1*, *Hox2*), *Hox3*, central class (*Hox4 *- *8*) and posterior class (*Hox9*-*15*) genes, are present in the annelid *Capitella teleta *[5], vertebrates and cephalochordates [15,16], which suggests that *Hox *genes were indeed organized in a cluster in the last common ancestor of protostomes and deuterostomes. However, it is still unclear how this cluster evolved, how many *Hox *genes were present in the 'core'-cluster of the ancestor before its expansion or how these *Hox *genes are related to their evolutionary sisters, the *ParaHox *genes [11,17-22].

### *Hox *and *Parahox *Evolution - Insight from Cnidarians and Acoels

The diversity of contexts in which *Hox *genes are utilized in bilaterian development illustrates the need to examine the deployment of these genes in broader evolutionary lineages. For example, it is still not known whether these genes were used to provide positional specific patterns of differentiation to all cells or if they were used to pattern specific compartments of organ systems such as the nervous system, axial mesoderm or the digestive tract. Furthermore, it is necessary to determine which tissue the ancestral *Hox *genes were patterning, since bilaterian *Hox *genes are involved in patterning different germ layers in different lineages [23,24]. In vertebrates, the same *Hox *genes are expressed in the neural tube and in paraxial mesoderm and have different anterior boundaries. This indicates that these genes were potentially co-opted independently for their roles in different tissues somewhere in the vertebrate lineage [25,26].

Cnidarians play an important role in unraveling the evolutionary origins of the *Hox *and *ParaHox *clusters, since they form the sister-group to the Bilateria [27] and do not appear to possess all *Hox *classes found in protostomes and deuterostomes. Recent investigations of the *Hox *gene complement in cnidarians (for example, corals, sea anemones, and jellyfish) have led to dramatically different interpretations of *Hox*/*ParaHox *gene evolution in the cnidarian-bilaterian ancestor [17,21,22,28,29]. One reason for this is the difficulty in establishing clear orthology assignments of cnidarian *Hox *and *ParaHox *genes to those of the Bilateria. In addition, lineage specific gene duplications have complicated attempts at reconstructing *Hox *gene evolution. Furthermore, studies of the expression patterns in representatives of the two branches of the Cnidaria - the anthozoans and medusozoans - suggest that their developmental function differs in both lineages [22,28]. However, the emerging consensus is that the last common cnidarian-bilaterian ancestor had one or two anterior and one posterior class gene that are clear orthologs to the bilaterian *Hox *genes and two *ParaHox *genes, namely a *Gsx *and a proto-*Xlox*/*Cdx *gene [22,28].

Since the cnidarian *Hox *complement appears to be much smaller than that of protostomes and deuterostomes, and also shows intra-taxon variation in the gene expression along the main body axes, bilaterian *Hox *cluster expansion remains unclear. Similarly, it is not clear when these genes first became involved in axial patterning and which structures first utilized *Hox *genes for their patterning. In order to gain more insight into the nature of the early bilaterian *Hox *genes and their role in body patterning, we investigated the expression patterns of the *Hox *gene complement in the acoels - a clade of relatively simple marine worms that posses an orthogonal nervous system and one mouth opening but lack an anus to the syncitial gut [30-33]. Morphological analyses [34] and molecular phylogenies using different genes suggest that acoels form an early branch in the Bilateria [35-41]. Phylogenomic studies had difficulty in placing acoels in the animal tree of life [27,42]. However, a recent phylogenomic study that uses 1487 genes places Acoela and Nemertodermatida - which together form a clade called Acoelomorpha [43] - as the sister group to all remaining Bilateria with high statistical support [44]. Morphological similarities between the stem cell system of acoels and platyhelminthes [36] are thus either ancestral traits of their last common ancestor or convergence [44].

Congruent with their phylogenetic position, acoels appear to possess only a subset of the *Hox *genes found in protostomes and deuterostomes, namely one anterior, one central and one posterior class gene, as well as an ortholog of the posterior *ParaHox *gene *Cdx *[45]. Nemertodermatida, the sister group of acoels, seem to have a similar subset of *Hox *genes belonging to the same three *Hox *classes [46]. A homeodomain survey on nemertodermatids revealed that, in addition to the *Cdx *gene present in acoels, a fragment of an *Xlox *ortholog has also been recovered [46]. These data suggest that the acoelomorph *Hox *complement is close to the predictions that have been made about the composition of the ancestral *Hox *cluster before its expansion in the remaining Bilateria [13,20]. As cnidarians, the sister group to the Bilateria, have no central class *Hox *gene, acoels are the pivotal group for reconstructing the origin and expansion of the central class genes and the possible roles of *Hox *genes in the evolution of bilaterian complexity.

We cloned orthologs of the three *Hox *gene classes and an ortholog of the *ParaHox *gene *Cdx *from the acoel *Convolutriloba longifissura *and investigated the embryonic and juvenile expression. We describe their deployment in relation to temporal and spatial collinear patterns and reconstruct their possible function in germ layer specification in the stem species of the Bilateria by comparing them with expression data from other bilaterian lineages.

## Results

### Gene orthology and composition of the acoel *Hox *genes

**Figure 1:**
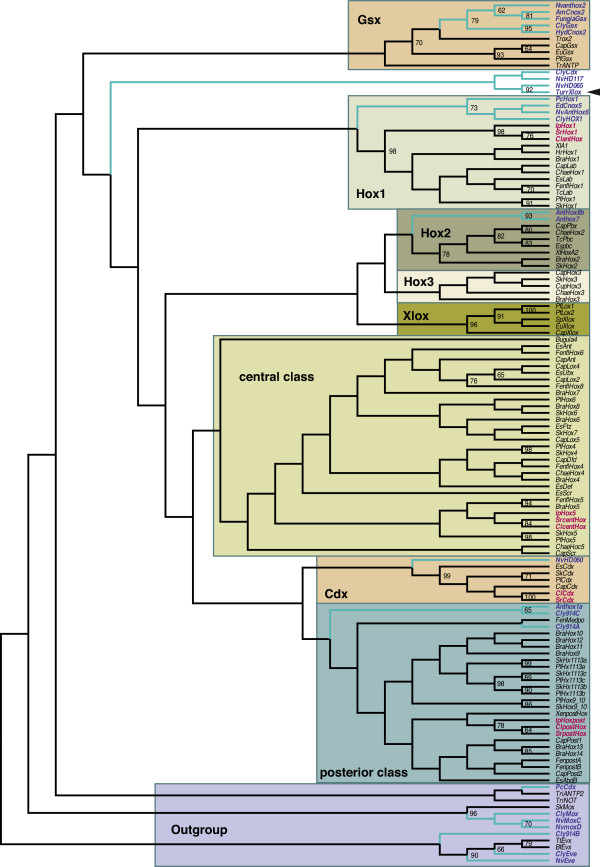
**Maximum likelihood phylogenetic analysis with cnidarian *Hox *orthologs**. Ingroup cnidarian sequences indicated in blue; *Hox *and *ParaHox *genes of acoels are indicated in red. The arrowhead points out the *Turritopsis *'Xlox' ortholog. Bootstraps above 60 are indicated in percentages. The alignment includes the flanking regions of Additional Table 1. Cl = *Convolutriloba; *Sr = *Symsagittifera*; Ip = *Isodiametra; *Nv = *Nematostella; *Cly = *Clythia; *Hyd = *Hydra; *Cap = *Capitella; *Tc = *Tribolium; *Fen = *Flaccisagitta; *Sk = *Saccoglossus; *Pf = *Ptychodera; *Bra = *Branchiostoma; *Chae = *Chaetopterous; *Tr = *Trichoplax; *Hr = *Haliotis; *Es = *Endeis; *Cup = *Cupiennius; *Xl = *Xenopus; *Pc = *Podocoryna; *Xen = *Xenoturbella; *Sp = *Strongylocentrotus*.

Gene orthology assignments of *Hox *genes are notoriously difficult to analyse because of a lack of phylogenetic signal contained in the 60 amino acids that compose the conserved homeodomain. The limited information results in extremely low support values for the basal branches and a high sensitivity of the tree topology to the methods and outgroups used in the analysis [17,21,22,28,47]. The inclusion of the cnidarian *Hox *and *ParaHox *gene sequences has led to distinctly different hypotheses about *Hox *and *ParaHox *gene evolution. Based on their phylogenetic position as a sister to protostomes and deuterostomes, acoels can provide an insight into the evolution of the *Hox *and *ParaHox *genes. We performed phylogenetic analyses of the *Hox *genes, using the homeodomain and flanking regions, and analysed the *Hox *gene composition of specific motifs that regulate the binding specificity of *Hox *genes in other metazoans (Figure 1, Additional file 1: Figure S1 and Table S1). Our primary goal was to establish the gene orthology to acoel *Hox *genes and we found that maximum likelihood (ML) analyses provided the most stable results. Tree topology was tested by conducting ML runs with and without cnidarian sequences (Figure 1, Additional file 1: Figure S1).

The results from both ML analyses of the acoel *Hox *genes were consistent with: one *C. longifissura Hox *gene grouping with the anterior (*Hox1/labial*); one with central (*Hox4/5*); and one with posterior *Hox *classes (Figure 1, Additional file 1: Figure S1). We have named these *ClantHox*, *ClcentHox*, and *ClpostHox*, respectively. The *Cdx *ortholog of *C. longifissura ClCdx *groups with the *Cdx *ortholog of the acoel *Symsagittifera roscoffensis *and other bilaterian *Cdx *orthologs (Figure 1, Additional file 1: Figure S1).

Orthology assignments are consistent in ML analyses, with or without cnidarian sequences. However, the topology of the branching of the individual paralog groups differs dramatically between both analyses. As in previous analyses, the basal branches receive no, or minor, support [17,21,22,28,47]. In the analysis that includes several hydrozoan and anthozoan *Hox *genes, the earliest branch is formed by the *ParaHox *gene *Gsx *(Figure 1). The exclusion of these genes led to an early branch of the *Hox1 *class genes (Additional file 1: Figure S1). These conflicting results have also been discovered in previous *Hox *gene analyses [22,28].

In neither analyses was the previously observed orthology assignments of the *ParaHox *genes *Gsx*, *Xlox *and *Cdx *to the *Hox *gene classes *Hox1*, *Hox3 *and *postHox *[12,19], respectively, have not been confirmed. If both gene clusters arose by a duplication of a proto-Hox cluster, the *ParaHox *genes would group as sisters to the corresponding *Hox *ortholog. However, this is not the case, since *ParaHox *genes either form separate branches (*Gsx *and *Xlox *in Figure 1) or their grouping with other *Hox *genes differs between the analyses. The cnidarian *Hox *and *ParaHox *orthologs form either the sister group to their bilaterian orthologs or form separate branches (*Cdx *and *postHox*, see Figure 1). In our analysis, the scyphozoan *Hox *gene which was previously assigned to the posterior *Hox *class (*CheHox9-14B*) groups with the *even-skipped *orthologs. We, therefore, cannot confirm that this is gene is a true *Hox *ortholog as has previously been stated [28]. The differences between these and previous analyses demonstrate the difficulties of *Hox *gene orthology assignment of cnidarian sequences and leave the question of the early origin of *Hox *and *ParaHox *genes unsolved. They do, however, indicate the significance of the cnidarian lineage for reconstructing early *Hox *gene evolution.

As the phylogenetic analyses of the homeodomain alone did not provide robust insight onto *Hox *gene orthology, we also compared motifs in N- and C-terminal flanking regions including the PBX-binding domain and its distance from the homeodomain in *Hox *and *ParaHox *orthologs (Additional file 1: Table S1). These motifs have been shown to play an important role in DNA-binding specificity and protein-protein interaction of *Hox *genes [23,48-51]. The PBX-motif is also present in non-*Hox *family genes (for example, in NK and LIM domain genes) [52] and is thus plesiomorphic for *Hox *genes. However, its presence, diagnostic motifs and distance (length of the 'linker') from the homeodomain has changed during the evolution of metazoan *Hox *genes and can thus provide phylogenetic information for the identification of *Hox *family information [52-57].

Overall, the acoel *Hox *sequences fit the expected pattern predicted in a previous study that analysed *Hox *gene structure [55]. The *anterior Hox *orthologs of *Isodiametra pulchra*, *Symsagittifera roscoffensis *and *Convolutriloba longifissura *contain a PBX-motif, which is a similar distance to the homeodomain (28/37/38 amino acids) compared to other bilaterian anterior class *Hox *genes (17-55 amino acids; Additional file 1: Table S1). The N-motif of the anterior *Hox *gene is similar to other bilaterian *Hox1 *orthologs (both share the Na-signature; see Additional file 1: Table S1). However, the C-motif of the anterior *Hox *ortholog in acoels is shorter than in all other *Hox *genes, with a stop-codon present close to the homeodomain (Additional file 1: Table S1).

The central class ortholog of acoels contains a PBX-motif that is close to the homeodomain (2 amino acids), as it is in the bilaterian orthologs of *Hox6*/*7 *(5-8 amino acids), but its C-motif has greater similarity to the bilaterian *Hox4/5 *orthologs. The acoel central class ortholog also shares similarities with the N-motif of bilaterian *Hox2*, *Hox3*, central class, *Gsx *and *Xlox *genes (all have the Nb motif). The posterior *Hox *ortholog of *C. longifissura *shares the PBX binding domain of ambulacrarian (hemichordates + echinoderms) and cephalochordate posterior *Hox *orthologs. The PBX-motif of posterior class *Hox *genes is located close to the N-terminal of the homeodomain and thus lacks the linker sequence found in the more anterior class *Hox *genes [52]. The N-motif of acoel posterior *Hox *genes also has posterior *Hox *specific residues (Nbx-motif; Additional file 1: Table S1).

The cnidarian putative *Hox *and *ParaHox *orthologs also share specific residues with their bilaterian orthologs. In the cases of the cnidarian anterior *Hox *and *ParaHox *(*Gsx*) orthologs, the motif pattern is conserved in relation to that of the bilaterian orthologs. A PBX-motif is present in the posterior *Hox *genes *Che9-14A *and *Che9-14C *of *Clytia *and in the *Nematostella Anthox1a*, which is located directly or close (a single amino acid) to the homeodomain which is similar to the bilaterian posterior *Hox *class genes. This supplies additional evidence that, contrary to previous suggestions, these genes are indeed posterior *Hox *genes [17,47]. The recently reported clear *Xlox *ortholog in the hydrozoan *Turritopsis dohrnii *[21] does not exhibit either the characteristic PBX binding domain or the similarities in the N-motif of bilaterian *Xlox *genes. This could be due to a secondary loss of these motifs in the hydrozoan lineage. However, an orthology of the gene with *Xlox *is not supported in our phylogenetic analysis, where it groups clearly with the previous described *Xlox*/*Cdx *ortholog (*NvHD065*) of *Nematostella *(Figure 1).

Although our phylogenetic analysis clearly assigns the acoel *Hox *genes to the anterior, central and posterior class, the internal grouping remains unclear. The lack of resolution and the lack of additional *Hox *genes in acoels cannot exclude that possibility that the three *Hox *genes found in acoels represent ancestral precursors of the bilaterian *Hox *cluster as has been suggested [20]. For this reason, we name the *Hox *genes according to their class (*Clant*-, *Clcent*-, *ClpostHox*) and do not make premature assignments to a specific sub-class of an extended bilaterian *Hox *complement. Our analysis suggests that a true anterior and posterior gene has been present in the bilaterian-cnidarian ancestor and that the central class genes are a novelty of the Bilateria. A *Hox3 *ortholog has not been found in cnidarians or acoels, so it appears to have evolved in the lineage to the common ancestor of protostome-deuterostome.

### Expression of *Convolutriloba longifissura *anterior class *Hox*

**Figure 2:**
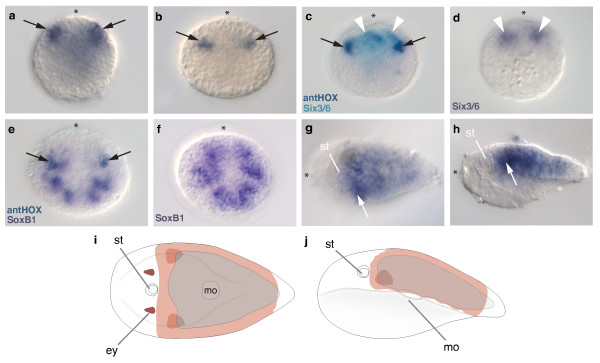
**Expression of *ClantHox *in embryos and juveniles of *C. longifissura***. (**a**) *ClantHox *expression in animal/anterior ectodermal cells in the postgastrula. (**b**) Bilateral domains of *ClantHox *expression located more internally in the animal hemisphere of the embryo during later development. (**c) **Double *in situ *hybridization of *ClantHox *(purple) with *ClSix3/6 *(turquoise), black arrows indicate the two *ClantHox *expression domains in the animal hemisphere. *ClSix3/6 *is found anterior to the *ClantHox *domain. Note that the *ClSix3/6 *staining is less localized than in (**d**) due to diffusion of the dye when using only 5-bromo-4chloro-3-indolyl-phosphate (BCIP) as substrate. (**d**) *ClSix3/6 *expression in the animal/anterior hemisphere. (**e**) Co-localization of the expression of *ClantHox *(area shown by a dotted line) with *ClSoxB1 *expression in the late postgastrula of *C. longifissura *both visualized with BCIP/nitro blue tetrazolium chloride (NBT). (**f**) *ClSoxB1 *expression clone of the same stage as (**e**). (**g**) Dorsal view on hatched juvenile showing expression of *ClantHox*. White arrow points at the higher expressing area in the post-statocyst commissure. (**h**) Lateral view in a hatched juvenile of *C. longifissura *with expression of *ClantHox *in the ectoderm and parenchyma. Arrow points at domain of commissural expression. (**i**) Summary of *ClantHox *expression is shown in orange, dorsal view, (**j**) lateral view. st = statocyst, ey = eye, mo = mouth opening. (**a - f**) Animal/anterior pole up; (**g - j**) anterior to the left.

The expression of the anterior class *Hox *gene in *C. longifissura *(*ClantHox*) starts after gastrulation when the embryo is composed of approximately 250 cells. It is expressed in two bilateral regions in the outermost cell layer in the animal hemisphere of the embryo (Figure 2a). Later, these domains of about 10 cells sink below the outermost cell layer (Figure 2b). Double *in situ *hybridizations with other marker genes allow us to determine the region of *ClantHox *expression. The acoel ortholog of *Six3/6, ClSix3/6 *(Figure 2c, d) is expressed in the future anterior region of the juvenile [58] anterior to *ClantHox *expression. *ClantHox *expressing cells (Figure 2e) co-express the pro-neural gene *ClSoxB1 *which suggests that these cells are neural with defined anterior-posterior boundaries (Figure 2i, j). Previous cell lineage experiments show that this domain is largely generated from the second duet micromeres [59]. In the juvenile, *ClantHox *is expressed posterior to the statocyst, extending to the end of the body but not at the posterior tip (Figure 2g, h). The strongest expression is found in a bilateral area directly posterior to the statocyst, which is likely to be comprised of descendants of cells expressing *ClantHox *during their early development (Figure 2c-f).

### Expression of *Convolutriloba longifissura *central class *Hox*

**Figure 3:**
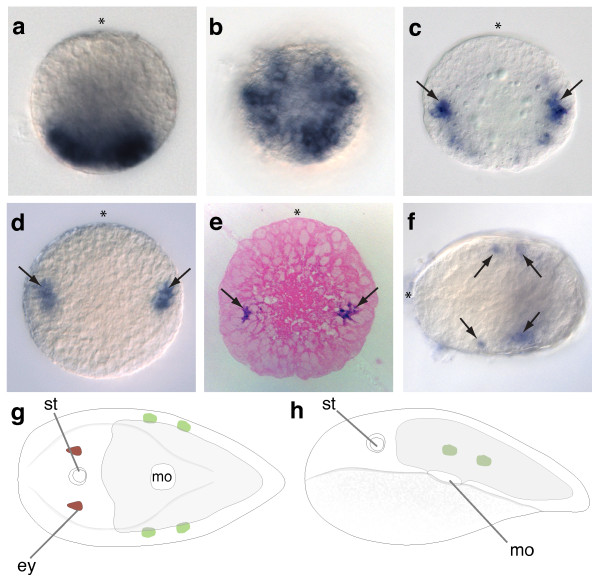
**Expression of *ClcentHox *in embryos of *C. longifissura***. (**a**) Lateral view on the earliest stage of detected *ClcentHox *expression. Expression is found at the vegetal pole at the site of the closed blastopore (**b**) Vegetal view at the stage of (**a**) showing that cells that express *ClantHox *surround the former blastopore. (**c**) Lateral view on postgastrula stage. The expression domain is below the ectoderm in two bilateral stripes with a stronger expression in the anterior cells (black arrows). (**d**) Later stage than (**c**) shows restriction of the expression domain of *ClcentHox *to a bilateral cluster of about a dozen cells. (**e**) Haematoxylin stained histological section of an embryo showing the *ClcentHox *expression below the outer ectodermal layer. (**f**) Pre-hatchling with bilateral *ClcentHox *expression domains that are localized in two separate domains along the anterior-posterior axis. (**g**) Summary of *ClcentHox *expression (green) in the juvenile from the dorsal side and left side (**h**) showing the two separate expression domains of *ClcentHox *on the left and right side. Asterisk indicates the animal/anterior pole of the embryo. (**g, h**) Animal to the left.

The expression of the *C. longifissura *central *Hox *class gene *ClcentHox *is detected by *in situ *hybridization shortly after gastrulation, about the same time as *ClantHox *(Figure 2a, 3a). Expression begins in the posterior part of the embryo (Figure 3a) in ectodermal cells surrounding the site of the closed blastopore (Figure 3b). During later development the expression domain is internalized and expands anteriorly on both sides, forming bilateral bands (Figure 3c). *ClcentHox *expression becomes refined to two internal bilateral spots of cells in the area lateral to the future position of the mouth (Figure 3d, e). The expression pattern is very similar to that of *ClantHox *at this stage (see Figure 2b). However, the anterior border of *ClcentHox *is more posterior. Like *ClantHox*, these bilateral cell patches are approximately the same size and are located below the epidermal surface. The identity of these expressing cells could not be determined because the cell types have not been differentiated at this stage. However, an endodermal fate can be excluded since the expression is not in the centre of the embryo, where the digestive syncytium will develop. The location below the outermost cell layer suggests a mesodermal or neural fate. However, these *ClcentHox*-expressing cells appear to be in the area of *ClSoxB1 *expression (Figure 2f, 3) which is thought to be involved in neural development in other animals [60]. This correlation with the expression patterns of 'neural' genes suggests that *ClcentHox *is expressed in neural precursor cells rather than mesodermally derived musculature. The pre-hatched juvenile shows strong expression in two small bilaterally paired domains that could be nerve cell bodies contributing to the orthogonal nervous system (Figure 3f-h).

### Expression of *Convolutriloba longifissura *posterior class *Hox*

**Figure 4:**
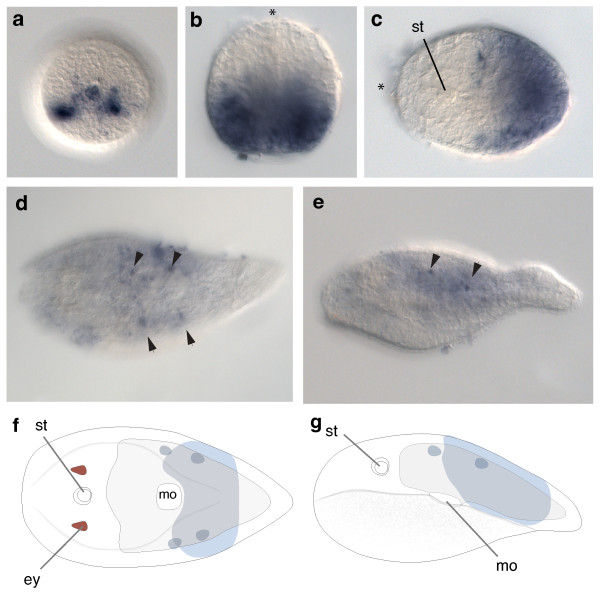
**Expression of *ClpostHox *in embryos and juveniles of *C. longifissura***. (**a**) Vegetal view on the site of gastrulation. *ClpostHox *expression begins at the vegetal pole of the postgastrula stage of *C. longifissura*. (**b**) Lateral view on the later embryo with the *ClpostHox *expression in the vegetal hemisphere. Asterisk indicates the animal pole. (**c**) *ClpostHox *expression is located in the posterior end in all three germ layers of pre-hatchlings. Cellular differentiation is indicated by the appearance of the statocyst (st). (**d**) Dorsal view on the hatched juvenile with fading *ClpostHox *expression. Localized centers of expression remain visible (black arrowheads). (**e**) Lateral view of a hatched juvenile of *C. longifissura *showing strong *ClpostHox *expression in individual cells in an area of weaker expression, anterior to the left. (**f**) + (**g**) Summary expression of *ClpostHox *(blue) in juveniles. Note that the posterior most tip of the animal is free of expression. Anterior to the left.

The expression of the ortholog of the posterior class *Hox *gene in *C. longifissura*, *ClpostHox*, starts at the same time as the other two *Hox *genes that is shortly after gastrulation (Figure 4a). Initially *ClpostHox *expression is detected in a restricted area of about 30 cells in the posterior region of the embryo, in the outermost cell layer. This domain extends over the vegetal hemisphere of the embryo (Figure 4b), which gives rise to the posterior part of the juvenile. The anterior border of the *ClpostHox *expression domain corresponds to the posterior border of the future mouth and thus overlaps with the posterior *ClcentHox *expressing domains. This expression of *ClpostHox *later expands to cells in all three germ layers, including the epidermis. However, during further development, as cell differentiation begins (for example. when the statocyst becomes visible), expression is down-regulated in the epidermis and endoderm (Figure 4c) and becomes restricted to a smaller lateral area below the outer cell layer with a weaker expression extending posteriorly (Figure 4d, e). The expression of *ClpostHox *persists in the juvenile and is found in four individual neural cells in a bilateral arrangement (Figure 4d-f). Expression in the hatchling is rather diffuse. The expression of *ClpostHox *is similar to that of the other two *Hox *orthologs (*ClantHox, ClcentHox*), suggesting that the gene is involved in neural patterning in the posterior region of the juvenile, but it may also have an early role in posterior patterning of all three germ layers.

### Expression of the *Convolutriloba longifissura ParaHox *gene *Cdx*

**Figure 5:**
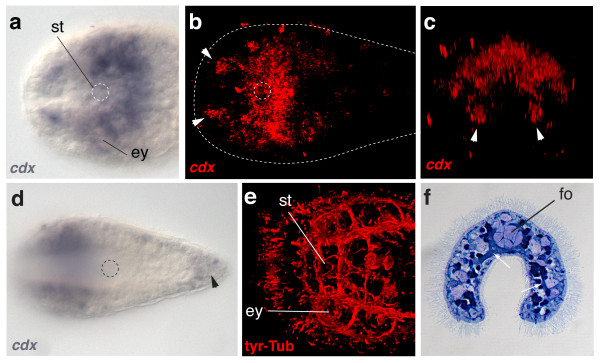
***ClCdx *expression in the juvenile nervous system of *C. longifissura***. **(a) **Dorsal view *ClCdx *expression in the juvenile head region. **(b) **Infra red-reflection microscopy of deep layers of the anterior part of the juvenile seen in (a). The outline of the animal is indicated with the dotted line. The white arrowheads indicate expression in the connectives extending to the anterior neural region. ey = eye, st = statocyst. **(c) **Frontal view of the image stack of (b). **(d) **Ventral view of the ectodermal expression of *ClCdx*. Expression flanks the latero-ventral nerve chords. The black arrowhead indicated the ectodermal expression in the region of the future gonopore. Mouth denoted by dotted circle **(e) **Dorsal view of a juvenile stained with anti-tyrosinated-tubulin antibody (red) showing a subset of the anterior commissures and connectives leading to the sensory cells surrounding the tip of the animal. **(f) **Nile-Blue stained histological cross section through the area anterior to the mouth, where *ClCdx *is expressed. White arrowheads point to nerve strands surrounding the gland cells of the frontal organ (fo) and extending nervous tissue in the ventral fold.

The expression pattern of *ClCdx *has been described previously [58,61] but here we focus on a detailed description of the anterior expression associated with the acoel nervous system and its relationship to other *Hox *gene expression patterns. *ClCdx *is expressed in the commissure posterior to the statocyst (Figure 5) and extends anteriorly and ventrally, following the paths of nerve tracks. *ClCdx *is also expressed in an area surrounding the eyes, which form direct connections to the brain commissure. The expression of *ClCdx *in the juvenile expands laterally to the posterior end, with a posterior domain in the ventral ectoderm that will form the male gonopore [58].

## Discussion

### Phylogenetic analyses and evolution of *Hox *genes

Orthology assignments of *Hox *genes are notoriously difficult due to the short amino acid sequence of the homeodomain. Previous comparisons of the homeodomains of *ParaHox *with that of the *Hox *genes of representative protostomes and deuterostomes revealed sequence similarities between the anterior, *Hox3 *and posterior *Hox *classes with that of *Gsx*, *Xlox *and *Cdx ParaHox *classes, respectively [12,19]. These findings led to the hypothesis of a early duplication of a 'proto-Hox' cluster composed of three or four proto-Hox genes which gave rise to two separate clusters of three/four *Hox *and three/four *ParaHox *genes [11,12,19]. However, recent analyses that included homeodomain genes from cnidarians suggest a rather different evolutionary origin of *Hox *and *ParaHox *genes in which a single 'proto-Hox' gene gave rise to a *Hox *and *paraHox *gene, forming an ancestral *Hox1 *and *Gsx *ortholog [22,28]. Tandem duplication events then led to the extension of *Hox *and *ParaHox *gene complement we find in the Bilateria [22]. This tandem-duplication hypothesis rejects the previously suggested duplication event of a 'proto-Hox' cluster that contained three, or even four, *Hox *genes (including a *Hox3 *ortholog) into a *Hox *and *paraHox *cluster and challenges the evolutionary relationship of posterior *Hox *to *Cdx *(posterior *ParaHox*) genes of *Xlox *to paralog group 3 *Hox *genes.

No study has yet found a *bona fide Hox3 *ortholog in either cnidarians or acoels and the recent report of an *Xlox *gene in a hydrozoan [21] seems to be an ortholog of the homeodomain gene *NvHD065 *in *Nematostella *(Figure 1). The finding of a clear *Gsx *ortholog in cnidarians [62] and *Trichoplax *[63], however, shows that either this *ParaHox *gene was lost in the acoel lineage or has not yet been found in the homeodomain gene surveys. Only whole genome sequencing can determine how many *Hox *genes are actually present in acoels and nemertodermatids.

To gain further insight into the phylogenetic relationships of acoel *Hox *genes we included both N- and C-terminus flanking regions of the homeodomain in our phylogenetic analysis and investigated the presence of conserved domains that have been shown to be responsible for the binding specificity. As in previous phylogenetic analyses of acoel *Hox *genes [5,45,64], the anterior class *Hox *orthologs of acoels groups as the sister to all remaining *Hox1*/*labial *genes, the central class *Hox *gene branches with *Hox4/5 *and the posterior *Hox *with the posterior class genes *Hox9-14*. At first glance, this suggests that a *Hox6/7 *gene has been present in the bilaterian ancestor and been lost in the acoel lineage. However, it does not exclude a tandem duplication event in which a proto central class gene gave rise to *Hox4/5 *and *Hox6/7 *and after which the one descendant (*Hox6/7*) deviated and became more in sequence with it than its sister (*Hox4/5*). The *Hox *complement of more basal branching lineages, such as cnidarians, could potentially deliver additional insight for reconstructing the *Hox *gene complement of the bilaterian ancestor.

The findings of the surveys for *Hox *and *ParaHox *genes in cnidarians suggest the presence of two *Hox *(anterior and posterior) and two clustered [65]*ParaHox *genes (*Gsx *and proto-*Cdx/Xlox*) in the last common ancestor of cnidarians and bilaterians. Together with the gene complement in acoels, the results favour a two-gene model of the evolution of the *Hox *genes from a proto-Hox cluster followed by several duplication events that gave rise to the central class and expansion within each class to give rise to the expanded Nephrozoan complement [13].

### The *Hox *orthologs in *Convolutriloba longifissura *are expressed in a spatially staggered pattern along the anterior-posterior body axis

Our results show, that the *Hox *genes of *C. longifissura *are expressed in a spatially staggered pattern along the anterior-posterior axis in later embryos and juveniles. As in most described bilaterians (except for hexapods and some polychaetes), orthologs of *Hox1/labial *are the most anteriorly-expressed genes of the *Hox *complement. In chordates this anterior border corresponds to the hindbrain, while in *Drosophila *this border corresponds to the tritocerebrum. *Hox1/labial *expression can be either restricted to a ring-like domain throughout the entire ectoderm (as in *Saccoglossus *[66]) or in a domain with a definite anterior boundary in neuroectodermal and mesodermal tissue that expands to the posterior end of the body (as in *Branchiostoma *[67] and vertebrates). In the protostomes investigated so far, *Hox1/labial *orthologs are expressed in a restricted anterior region [5,68-70], starting in two bilateral domains (in the polychaetes *Capitella, Nereis, Chaetopterus*), which is similar to the pattern we describe for *ClantHox *in *C. longifissura*. In the juvenile, the most anterior parts of the acoel nervous system anterior to the statocyst are free of *ClantHox *expression, which is similar to the conditions found in protostomes and deuterostomes (for example, *Haliotis*, *Platynereis*, *Capitella*, *Saccoglossus*, *Branchiostoma, Drosophila, Chaetopterous*). In both protostomes and deuterostomes, the most anterior part of the nervous system lacks any *Hox *gene expression but usually expresses the gene *Six3/6 *[71], which is also the case in *C. longifissura *(Figure 3).

Posterior to the bilaterally restricted patches of *ClantHox *expression, similar domains of the central class *Hox *gene (*ClcentHox*) are expressed in the acoel embryo. Behind these *ClcentHox *expressing patches of cells, the expression domain of the single posterior class *Hox *ortholog, *ClpostHox *begins and it extends to the posterior end of the embryo. This indicates that the spatial colinearity of *Hox *gene expression was present in the last common ancestor of the Bilateria and that the system that regulates the spatial colinearity was maintained, despite the expansion of the *Hox *cluster during bilaterian evolution.

The boundaries of expression of the three *Hox *genes in the un-segmented body of the acoel do not correspond to any overt morphological boundaries as is found in the segments of arthropods, annelids and in the vertebrae of mice. However, the anterior border of the *ClantHox *expression corresponds to the 'brain' commissure behind the statocyst and the patch-like expression of *ClcentHox *and *ClpostHox *seem to correspond to specific neural precursors in the orthogonal nervous system (Figure 2, 3, 4). The mouth opening in acoels is formed late in development [72] and its position corresponds to the border just anterior to the expression of the posterior *Hox *ortholog (Figure 4).

### Expression of *Hox *orthologs in *Convolutriloba longifissura *begins simultaneously after gastrulation and shows no temporal colinearity

**Figure 6:**
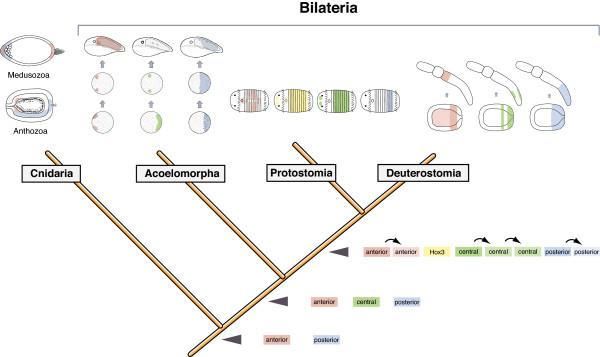
**Evolutionary scenario of *Hox *gene expression evolution in the Bilateria**. The two gene model of *Hox *gene evolution is hypothesized and its stepwise extension in the evolution of the Bilateria. The cnidarian-bilaterian ancestor possessed two clear *Hox *genes which are expressed ambiguously in the taxon cnidaria. One major branch, the Anthozoa, express the anterior ortholog in the pharynx and the posterior orthologs along the oral-aboral axis. The other major branch (Medusozoa) the posterior ortholog is expressed in the oral pole, while the anterior ortholog is expressed at both ends of the body. Based on the data of *Hox *gene expression of the acoelomorph *C. longifissura*, the three *Hox *genes are expressed in a spatial staggered pattern along the anterior-posterior axis. The expression is switched on simultaneously in the acoel, which excludes temporal colinearity. The spatial staggered expression however, is ancestral for the Bilateria lineage. The protostome *Hox *expression is represented by the annelid *Capitella teleta *[5], the deuterostome expression by the hemichordate *Saccoglossus *[71]. The acoel expression shares the blastoporal expression of the central and posterior class orthologs in *Saccoglossus*, the anterior ortholog is not expressed.

Our study of the early expression patterns of the *Hox *gene complement of *C. longifissura *shows that all three *Hox *genes are expressed almost simultaneously at a time after gastrulation when the embryo is composed of about 250 cells (Figure 6). Our observations suggest that there is little temporal colinearity in this acoel species, although we cannot exclude a slightly temporal staggered expression of one or two cell cycles. We interpret our findings as a lack of temporal colinearity of expression, unlike that present in animals which have an intact, organized *Hox *cluster, such as vertebrates [8], cephalochordates [67], the polychaete *Capitella teleta *[5], and some insects [73]. Those examples in which the *Hox *cluster has been dispersed, split or disorganized, such as in *Drosophila *([74], *C. elegans *[75], *Ciona *[76], *Saccoglossus *[66], *Oikopleura *[77] and *Strongylocentrotus *[78], lack temporal colinearity of *Hox *expression. Thus, temporal colinearity of *Hox *expression seems to be associated with a functionally intact *Hox *cluster [10].

The lack of dramatic temporal staggering of the *Hox *gene expression in acoels can be interpreted in two ways. Given the consensus view of the presence of a complete *Hox *cluster with temporal and spatial colinearity at the base of the Bilateria, the lack of temporal colinearity in acoels would suggest that an ancestral *Hox *cluster has dispersed in the acoel lineage. The reasons for a possible *Hox *cluster dispersion in acoels are not particularly clear. Explanations used in other taxa (for example, *Drosophila*, nematodes, *Oikopleura*) such as rapid development, short generation time or the emergence of a rigid stereotyped cleavage pattern with precocious specification of cell fates [4] do not appear to be applicable in acoels. Although acoel development displays a stereotyped cleavage programme, it is not particularly rapid (occurs in 4-5 days) and the embryos and adults show a high degree of regulative capacity [79]. Most adult acoels are long lived, capable of regeneration and can even reproduce asexually.

Assuming the presence of an integrated *Hox *cluster in the last common ancestor of these animals, it has been suggested that the concerted mechanism that controls the temporal expression prevents the ancient cluster from its evolutionary dispersion [10,18,80,81]. Recent evidence shows, that the temporal and spatial patterning of *Hox *expression is controlled by distinct mechanisms [82-84], which might explain why spatial colinearity is maintained even during cluster disintegration [77].

Since the expression of the *Hox *genes in acoels are turned on simultaneously, another explanation for the lack of temporal colinearity could be that the elements that control a temporal staggered expression has evolved in the nephrozoan lineage in combination with the expansion of the cluster. The temporally staggered regulation of *Hox *clusters would then be linked by the expansion of the cluster to a larger number of genes and/or a larger genomic region in the course of the *Hox *cluster evolution of the protostome-deuterostome ancestor [85]. However, a recent analysis using sequencing of bacterial artificial chromosome libraries and fluorescent *in situ *hybridization in an acoel species suggests a dispersed cluster of *Hox *genes [86].

### The *Hox *gene expression in acoels is correlated with neural development in an axial fashion

Our results suggest, that the *Hox *genes in the acoel *C. longifissura *might play a major role in the axial patterning of the nervous system. The simultaneous onset of the *Hox *genes in the ectoderm followed by the internalization of the expressing cells reflects the internal migration of putative neural precursor cells and is consistent with the results of fate mapping that showed that the nervous system is formed by descendants of the first, second and third duet ectodermal micromeres [59]. The co-expression of *ClantHox *with the *Convolutriloba *ortholog of the bilaterian proneural gene *SoxB1 *[87] is consistent with the previously described patterns in other bilaterians and suggests that *ClantHox *is responsible for the patterning anterior subsets of the nervous system. The overlapping expression of *ClcentHox *with *ClSoxB1 *also suggests a function of *ClcentHox *in the formation of neural precursor cells in the mid-body region. In contrast, *ClpostHox *expression is found in a broad posterior domain that includes ectodermal, endodermal and, possibly, mesodermal cells suggesting that it may be involved in patterning more than just the nervous system. However, *ClpostHox *expression in the juvenile seems to be more restricted to cells that contribute to the nervous system.

The strong correlation of the acoel *Hox *gene expression with the nervous system development supports the idea of the ancestral role of *Hox *genes in the anterior-posterior patterning and regionalization of the central nervous system. This has been hypothesized since the discovery of *Hox *gene expression in protostomes (leech [88-91], *C. elegans*: [92]*Drosophila*: [93]) and deuterostomes [24,94,95]. Recent studies in the polychaete annelids *Capitella *[5], *Chaetopterus *[69], nereids [70], the molluscs *Haliotis *[68]*Euprymna *[96] and the deuterostomes *Saccoglossus *[66], *Branchiostoma *[67]*Ciona *[97,98] and *Oikopleura *[77] all found that *Hox *genes are expressed in neural tissue [99]. As the nervous system plays such important part in the body plan organization in bilaterians, the ancestral *Hox *gene expression in the central nervous system may have been co-opted for the more complex patterning of additional tissues along the anterior-posterior axis, such as paraxial mesoderm, paired appendages and genitalia [100].

The staggered spatial expression of *Hox *genes in protostome and deuterostome centralized nervous systems, has also been used to homologize neuroanatomical structures (for example, the tritocerebrum of *Drosophila *with the hindbrain of chordates) [101,102]. However, our results from the acoel show that a similar staggered anterior-posterior expression of *Hox *genes was already present in the nervous system of the last common bilaterian ancestor with a much simpler neural organization. This indicates that metazoan animals appeared to have a general molecular blueprint for axial organization that did not specify the distinct morphological complexity of the final structures. Therefore, the homologization of more complex brain structures between protostomes and deuterostomes on the basis of *Hox *gene expression is inappropriate. This interpretation is supported by broad comparative studies utilizing the diversity of animal taxa, many of which lack complex brains but possess a similar arrangement of *Hox *expression, such as the hemichordate *Saccoglossus *[66,103] and the annelid *Capitella *[5].

### Early blastoporal expression suggests an early role of *Hox *genes in ectodermal patterning of acoels

Two of the three *Hox *genes of *C. longifissura *- the central and the posterior class *Hox *gene - are expressed at the site of gastrulation. The early onset of *ClcentHox *at the area of the former blastopore is similar to that found for *Hox5 *in the hemichordate *Saccoglossus *[66] and *Branchiostoma *[67] and for *Hox4 *and *Hox5 *in the polychaete *Nereis *[70] and *Chaetopterus *[69]. It remains unclear how the specific cell divisions and expression pattern correlates to the spatially very restricted position of *ClcentHox *expression in two bilateral clusters lateral to the position of the future mouth during later development. Both *ClcentHox *and *ClpostHox *are first expressed in the posterior ectoderm in an overlapping manner, before becoming restricted to neural cell fates when the staggered pattern of the expression is established at later stages of development. This restriction is first observed in the expression pattern of *ClcentHox *followed by the restriction of *ClpostHox *expression. However, the early expression of these two *Hox *genes at the blastopore suggests a different function from the later neural expression. A similar biphasic temporal expression is also found in chick development, where the early function of the *Hox *genes seems to be in axial specification of the gastrulating cells at the primitive streak [104]. Perhaps the *Hox *genes are involved with chromatin modification, which creates a 'memory' for the future fates, even when the gene is no longer expressed [104]. A similar mechanism could be present in the acoels, polychaetes and hemichordates and needs further investigations.

### The expression of the *ParaHox Cdx *ortholog in *C. longifissura*

The role of *Cdx *expression in acoels, in hindgut evolution and the expression along the anterior-posterior axis in different germ layers have already been discussed [58,61]. The expression of *ClCdx *in the anterior brain-commissures and connectives that extend to the ventral fold and the anterior sensory cells is very similar to the expression pattern found in the brain of the polychaete *Capitella teleta *[105]. Interestingly, *Cdx *does not seem to be expressed in the brain of nereid polychaetes [106,107] which indicates intra-taxon variability. However, other bilaterians investigated also lack *Cdx *expression in the anterior neural structures but show *Cdx *expression in neural and endodermal tissue [108]. Thus, it remains unclear how far the brain expression in *Capitella teleta *reflects evolutionary ancestry with the acoel neural expression.

The findings that, in several bilaterians, *ParaHox *genes are expressed associated with the digestive system led to the hypothesis that *Gsx*, *Xlox *and *Cdx *are responsible for the AP patterning of a through gut in the hypothetical Urbilaterian [109]. However, this pattern is not consistent throughout the Bilateria [reviewed in [105]]. The expression of *Cdx *in the posterior ectoderm in most bilaterian lineages, which often forms the ectodermal hindgut, seems to be highly conserved [110]. In the acoels and nemertodermatids the posterior ectoderm forms the male gonoduct of the adult, which is also composed of at least a part of ectoderm. This suggests that the posterior ectodermal expression was co-opted for hindgut formation in different animal lineages [58,111].

Our survey of *Hox *and *ParaHox *genes in the acoel *C. longifissura *did not reveal any *ParaHox *genes other than *Cdx*. This could indicate a loss of additional paralogs or highly divergent homeodomain sequences which could not be recovered with our degenerate polymerase chain reaction (PCR) approach. The fact that an ancestral epithelial digestive system, as it is present in nemertodermatids and cnidarians, has been reduced the to a syncytium in the acoel lineage [33] could explain a loss of these genes from the genome. Since the sister group Nemertodermatida possesses an epithelial gut and an *Xlox *ortholog, expression studies of these genes in these worms may indicate that there was an ancestral role of the other *ParaHox *genes in bilaterian gut patterning.

## Conclusion

Our results of the study of the embryonic expression patterns of the *Hox *genes in a representative of the likely earliest branch of the Bilateria, the acoelomorphs, show that the small *Hox *gene complement is expressed in a staggered pattern along the anterior-posterior body axis (Figure 6). This suggests that the spatial expression was regulated by an ancestral regulatory system in the last common ancestor of all bilaterians. However, the simultaneous initiation of *Hox *gene expression in the acoel shows a lack of the temporal colinearity that is present in some bilaterian lineages, perhaps due to a breakdown of genomic clustering. Our description of the biphasic expression of the *Hox *genes in the embryo of the acoel *C. longifissura *not only suggests a early function during gastrulation but that the later co-localization with pro-neural genes indicates the ancestral role of the *Hox *genes in nervous system patterning which was co-opted for the patterning of other tissue layers and organ systems later in the bilaterian evolution.

## Methods

### Material

Gravid adult acoels of the hermaphroditic species *Convolutriloba longifissura *were collected from the sea water tables of the Kewalo Marine Laboratory (Oahu, Hawaii) and reared in glass fingers bowls of filtered seawater in constant light. Egg clusters composed of 25 - 70 zygotes were harvested the morning after collection. The culture of this and related *Convolutriloba *species has been described elsewhere [112]. Embryos were staged and grown in petri-dishes in 0.22 μm Millipore filtered seawater. *Convolutriloba longifissura *embryos develop directly to juvenile adults with no larval phase, hatching 4 - 5 days after ovoposition at 25°C.

### Gene amplification

We used standard degenerate primers F: 5-GARYTNGARAARGARTT-3 (ELEKEF) and R: 5-CKNCKRTTYTGRAACCA-3 (WFQNRR) for *Hox *gene amplification from *Convolutriloba longifissura *genomic DNA. The search was extended with primers against specific *Hox *and *ParaHox *genes (F1cHOX 5-MGNACNMGNACNGCNTA-3, F2cHOX 5-ACNGCNTAYACNMGNTTY-3, GsxF1 5'-ATG YCG CGW TCW TTT YTS RTG GA-3', GsxF2 5'-TTT YTS RTG GAT TCN YT-3', XloxF1 5-GAYGARAAYAARMGNACNMGNAC-3) and sequencing of 325 clones only led to the three Hox and one ParaHox gene. *ClSoxB1 *fragments were amplified from cDNA of mixed embryonic stages using the degenerate primers SoxBF1 5'- GTNAARMGNCCNATGAAYGC-3', SoxBF2 5'-GGNCARMGNMGNAARATG-3', SoxBR1 5'-TTNGKYTTNCKNCKNGG-3', SoxBR2 5'-TAYTTRTARTCNGGRTGYTC-3'. Gene ends were recovered using RACE PCR (BD Smart RACE kit, Clontech) in both 5' and 3' direction. For all fragments pGEM-T-easy (Promega) was used as the cloning vector. The gene sequences are deposited in GeneBank with the following accession numbers: *ClSoxB1*: GQ487528, *ClantHox *GQ487529, *ClcentHox *GQ487530, *ClpostHox *GQ487531.

### Phylogenetic analysis

To determine gene orthology of the *ClSoxB1 *gene, phylogenetic analysis using Bayesian inference was conducted (mixed model option and 3,000,000 generations sampled every 1000 generations and 4 chains). A maximum likelihood analysis using the software PhyML [113] with 3000 bootstraps was conducted in order to analyse the gene orthology of the *Hox *genes amplified in this analysis, along with published sequences from the acoels *Symsagittifera roscoffensis *and *Isodiametra pulchra*. In addition to the homeodomain, 5' and 3' flanking regions were incorporated in the alignment, including the PBX domain when present (see Additional file 1: Table S1). The software MUSCLE [114] was used for the alignment and corrected manually. The best protein evolution model (RTRev) was determined with the software Protest. Extended *Hox *class genes [115] were used as outgroup. [The nexus files are available on request at andreas.hejnol@sars.uib.no].

### Fixation

Embryos of known developmental stages were fixed in 3.7% formaldehyde in seawater for 3 hours at 4°C. Embryos were washed in PTw (PBS + 0.1% Tween 20) with several changes for at least 2 hours to dissolve the red pigment. After a 5-minute wash with distilled water to remove salt, embryos and juveniles were dehydrated by several washes in 100% methanol. Hatchlings and embryos were stored in separate tubes at -20°C for several months and rehydrated for whole mount *in situ *hybridizations.

### Whole mount *In situ *hybridization

*In vitro *transcribed (Ambion Megascript T7 or SP6 kit) DIG-labelled antisense *in situ *probes were made from PCR amplified gene fragments (*ClCdx *1155 bp, *ClcentHox *855 bp, *ClantHox *1006 bp, *ClpostHox *900 bp, *ClSoxB1 *1450 bp). The whole mount *in situ *hybridization protocol has been previously described and is available online (http://www.natureprotocols.com/2008/09/18/in_situ_protocol_for_embryos_a.php). Double *in situ *hybridizations were carried out using both DIG-labelled and fluorescein-labeled riboprobes. DIG-labelled riboprobes were detected colourimetrically with NBT/BCIP substrates. After the first colour reaction, the alkaline-phosphatase of the anti-DIG antibody was heat-inactivated by incubation for 30 minutes at 60°C, followed by incubation with 0.1 M glycine-HCl (pH 2.2). Fluorescein-labelled riboprobes were detected using only 5-bromo-4-chloro-3-indolyl-phosphate (BCIP). However, this precipitate is more diffuse than the product of the reaction using nitro blue tetrazolium chloride (NBT)/BCIP as a substrate. Additional comparisons of the expression patterns of both genes were made using NBT/BCIP simultaneously for both probes.

Embryos were mounted in 70% glycerol and the expression patterns were documented using an Axiocam high resolution camera on an Axioscope mot2 plus with Axiovision (Zeiss, Inc, VA, USA) software. The precipitate of the NBT/BCIP reaction reflects infrared light and stacks of expression patterns for three dimensional reconstructions were imaged using NBT/BCIP IR reflection microscopy with a Zeiss LSM 510 confocal microscope [116].

### Histology

Embryos were embedded in glycol methacrylate (Technovit^® ^7100) after *in situ *hybridization according to the manufactures (Heraeus Kulzer, Germany) protocol, and sectioned with a microtome with glass knives at the Pacific Biosciences Research Center (University of Hawaii, Honolulu) microscopy facility or with a sliding microtome at the Institute for Biology (Humboldt-University Berlin). Sections were stained with haematoxylin or nile blue using standard histological protocols.

### Immunohistochemistry

To label the serotonergic nervous system we used an antibody against tyrosinated-tubulin (Sigma, USA) raised in mouse in a concentration 1:200 using a standard neural antibody staining protocol [117] and visualized using a Cy3 labelled anti-mouse secondary anti-body (Jackson Laboratories, USA).

## Abbreviations

A-P: anterior-posterior; ML: maximum likelihood; PCR: polymerase chain reaction; BCIP: 5-bromo-4-chloro-3-indolyl-phosphate; NBT: nitro blue tetrazolium chloride.

## Authors' contributions

AH and MQM wrote the manuscript and designed the study. AH performed the experiments. Both authors read and approved the final manuscript.

## Supplementary Material

Additional file 1 **Additional figures and table**. Additional Data Figure S1 - Phylogenetic analysis of *Hox *and *ParaHox *genes that excluded most cnidarian orthologs. Additional Data Figure S2 - Bayesian orthology assignment of *ClSoxB1*. Additional Data Table S1 - Motif comparisons of acoel Hox genes with that of other bilaterians and cnidarians. Click here for file

## References

[B1] Akam M (1987). The molecular basis for metameric pattern in the *Drosophila *embryo. Development.

[B2] Burke AC, Nelson CE, Morgan BA, Tabin C (1995). Hox genes and the evolution of vertebrate axial morphology. Development.

[B3] Akam M (1989). Hox and HOM: homologous gene clusters in insects and vertebrates. Cell.

[B4] Duboule D (2007). The rise and fall of Hox gene clusters. Development.

[B5] Fröbius AC, Matus DQ, Seaver EC (2008). Genomic organization and expression demonstrate spatial and temporal *Hox *gene colinearity in the lophotrochozoan *Capitella *sp. I. PLoS One.

[B6] Gaunt SJ (1988). Mouse homeobox gene transcripts occupy different but overlapping domains in embryonic germ layers and organs: a comparison of Hox-3.1 and Hox-1.5. Development.

[B7] Minguillon C, Gardenyes J, Serra E, Castro LF, Hill-Force A, Holland PW, Amemiya CT, Garcia-Fernandez J (2005). No more than 14: the end of the amphioxus Hox cluster. Int J Biol Sci.

[B8] Izpisua-Belmonte JC, Falkenstein H, Dolle P, Renucci A, Duboule D (1991). Murine genes related to the *Drosophila *AbdB homeotic genes are sequentially expressed during development of the posterior part of the body. Embo J.

[B9] Dolle P, Izpisua-Belmonte JC, Falkenstein H, Renucci A, Duboule D (1989). Coordinate expression of the murine Hox-5 complex homoeobox-containing genes during limb pattern formation. Nature.

[B10] Monteiro AS, Ferrier DE (2006). Hox genes are not always Colinear. Int J Biol Sci.

[B11] Ferrier DE, Holland PW (2001). Ancient origin of the Hox gene cluster. Nat Rev Genet.

[B12] Brooke NM, Garcia-Fernandez J, Holland PW (1998). The ParaHox gene cluster is an evolutionary sister of the Hox gene cluster. Nature.

[B13] Garcia-Fernàndez J (2005). Hox, ParaHox, ProtoHox: facts and guesses. Heredity.

[B14] Garcia-Fernàndez J (2005). The genesis and evolution of homeobox gene clusters. Nat Rev Genet.

[B15] Hoegg S, Meyer A (2005). Hox clusters as models for vertebrate genome evolution. Trends Genet.

[B16] Holland LZ, Albalat R, Azumi K, Benito-Gutierrez E, Blow MJ, Bronner-Fraser M, Brunet F, Butts T, Candiani S, Dishaw LJ (2008). The amphioxus genome illuminates vertebrate origins and cephalochordate biology. Genome Res.

[B17] Chourrout D, Delsuc F, Chourrout P, Edvardsen RB, Rentzsch F, Renfer E, Jensen MF, Zhu B, de Jong P, Steele RE (2006). Minimal ProtoHox cluster inferred from bilaterian and cnidarian Hox complements. Nature.

[B18] Ferrier DE, Minguillon C (2003). Evolution of the Hox/ParaHox gene clusters. Int J Dev Biol.

[B19] Kourakis MJ, Martindale MQ (2000). Combined-method phylogenetic analysis of Hox and ParaHox genes of the metazoa. J Exp Zool.

[B20] Lanfear R, Bromham L (2008). Statistical tests between competing hypothesis of Hox cluster evolution. Syst Biol.

[B21] Quiquand M, Yanze N, Schmich J, Schmid V, Galliot B, Piraino S (2009). More constraint on *ParaHox *than *Hox *gene families in early metazoan evolution. Dev Biol.

[B22] Ryan JF, Mazza ME, Pang K, Matus DQ, Baxevanis AD, Martindale MQ, Finnerty JR (2007). Pre-bilaterian origins of the Hox cluster and the Hox code: evidence from the sea anemone, *Nematostella vectensis*. PLoS ONE.

[B23] Chauvet S, Merabet S, Bilder D, Scott MP, Pradel J, Graba Y (2000). Distinct hox protein sequences determine specificity in different tissues. Proc Natl Acad Sci USA.

[B24] Pearson JC, Lemons D, McGinnis W (2005). Modulating Hox gene functions during animal body patterning. Nat Rev Genet.

[B25] Frasch M, Chen X, Lufkin T (1995). Evolutionary-conserved enhancers direct region-specific expression of the murine *Hoxa-1 *and *Hoxa-2 *loci in both mice and *Drosophila*. Development.

[B26] Kwan CT, Tsang SL, Krumlauf R, Sham MH (2001). Regulatory analysis of the mouse *Hoxb3 *gene: multiple elements work in concert to direct temporal and spatial patterns of expression. Dev Biol.

[B27] Dunn CW, Hejnol A, Matus DQ, Pang K, Browne WE, Smith SA, Seaver E, Rouse GW, Obst M, Edgecombe GD (2008). Broad phylogenomic sampling improves resolution of the animal tree of life. Nature.

[B28] Chiori R, Jager M, Denker E, Wincker P, Da Silva C, Le Guyader H, Manuel M, Queinnec E (2009). Are Hox genes ancestrally involved in axial patterning? Evidence from the hydrozoan *Clytia hemisphaerica *(Cnidaria). PLoS ONE.

[B29] Kamm K, Schierwater B (2006). Ancient complexity of the non-Hox ANTP gene complement in the anthozoan *Nematostella vectensis *: implications for the evolution of the ANTP superclass. J Exp Zoolog B Mol Dev Evol.

[B30] Baguñá J, Riutort M (2004). The dawn of bilaterian animals: the case of acoelomorph flatworms. Bioessays.

[B31] Bourlat SJ, Hejnol A (2009). Acoels. Curr Biol.

[B32] Hyman LH (1951). The Invertebrates. Vol II. Platyhelminthes and Rhynchocoela.

[B33] Smith J, Tyler S, Conway Morris S, George JD, Gibson R, Platt HM (1985). The acoel turbellarians: kingpins of metazoan evolution or a specialized offshoot?. The origins and relationships of lower invertebrates.

[B34] Haszprunar G (1996). Plathelminthes and Plathelminthomorpha - paraphyletic taxa. J Zool Syst Evol Res.

[B35] Carranza S, Baguñà J, Riutort M (1997). Are the Platyhelminthes a monophyletic primitive group? An assessment using 18S rDNA sequences. Mol Biol Evol.

[B36] Egger B, Steinke D, Tarui H, De Mulder K, Arendt D, Borgonie G, Funayama N, Gschwentner R, Hartenstein V, Hobmayer B (2009). To be or not to be a flatworm: the acoel controversy. PLoS ONE.

[B37] Jondelius U, Larsson K, Raikova O (2004). Cleavage in *Nemertoderma westbladi *(Nemertodermatida) and its phylogenetic significance. Zoomorphology.

[B38] Paps J, Baguñà J, Riutort M (2009). Lophotrochozoa internal phylogeny: new insights from an up-to-date analysis of nuclear ribosomal genes. Proc Biol Sci.

[B39] Ruiz-Trillo I, Paps J, Loukota M, Ribera C, Jondelius U, Baguñà J, Riutort M (2002). A phylogenetic analysis of myosin heavy chain type II sequences corroborates that Acoela and Nemertodermatida are basal bilaterians. Proc Natl Acad Sci USA.

[B40] Ruiz-Trillo I, Riutort M, Littlewood DTJ, Herniou EA, Baguñà J (1999). Acoel flatworms: earliest extant bilaterian Metazoans, not members of Platyhelminthes. Science.

[B41] Wallberg A, Curini-Galletti M, Ahmadzadeh A, Jondelius U (2007). Dismissal of Acoelomorpha: Acoela and Nemertodermatida are separate early bilaterian clades. Zoologica Scripta.

[B42] Philippe H, Brinkmann H, Martinez P, Riutort M, Baguñà J (2007). Acoel flatworms are not platyhelminthes: evidence from phylogenomics. PLoS ONE.

[B43] Ehlers U (1985). Das phylogenetische System der Plathelminthes.

[B44] Hejnol A, Obst M, Stamatakis A, Ott M, Rouse GW, Edgecombe GD, Martinez P, Baguñà J, Bailly X, Jondelius U (2009). Assessing the root of bilaterian animals with scalable phylogenomic methods. Proc R Soc Sci B.

[B45] Cook CE, Jiménez E, Akam M, Saló E (2004). The Hox gene complement of acoel flatworms, a basal bilaterian clade. Evol Dev.

[B46] Jiménez-Guri E, Paps J, García-Fernàndez J, Saló E (2006). Hox and ParaHox genes in Nemertodermatida, a basal bilaterian clade. Int J Dev Biol.

[B47] Kamm K, Schierwater B, Jakob W, Dellaporta SL, Miller DJ (2006). Axial patterning and diversification in the cnidaria predate the Hox system. Curr Biol.

[B48] Ekker SC, Jackson DG, von Kessler DP, Sun BI, Young KE, Beachy PA (1994). The degree of variation in DNA sequence recognition among four *Drosophila *homeotic proteins. Embo J.

[B49] Gebelein B, Culi J, Ryoo HD, Zhang W, Mann RS (2002). Specificity of *Distalless *repression and limb primordia development by abdominal Hox proteins. Dev Cell.

[B50] Merabet S, Hudry B, Saadaoui M, Graba Y (2009). Classification of sequence signatures: a guide to Hox protein function. Bioessays.

[B51] Merabet S, Kambris Z, Capovilla M, Berenger H, Pradel J, Graba Y (2003). The hexapeptide and linker regions of the AbdA Hox protein regulate its activating and repressive functions. Dev Cell.

[B52] In der Rieden PM, Mainguy G, Woltering JM, Durston AJ (2004). Homeodomain to hexapeptide or PBC-interaction-domain distance: size apparently matters. Trends Genet.

[B53] de Rosa R, Grenier JK, Andreeva T, Cook CE, Adoutte A, Akam M, Carroll SB, Balavoine G (1999). Hox genes in brachiopods and priapulids and protostome evolution. Nature.

[B54] Morgan R, In der Rieden P, Hooiveld MH, Durston AJ (2000). Identifying HOX paralog groups by the PBX-binding region. Trends Genet.

[B55] Ogishima S, Tanaka H (2007). Missing link in the evolution of Hox clusters. Gene.

[B56] Passamaneck YJ, Halanych KM (2004). Evidence from Hox genes that bryozoans are lophotrochozoans. Evol Dev.

[B57] Telford MJ (2000). Turning Hox "signatures" into synapomorphies. Evol Dev.

[B58] Hejnol A, Martindale MQ (2008). Acoel development indicates the independent evolution of the bilaterian mouth and anus. Nature.

[B59] Henry JQ, Martindale MQ, Boyer BC (2000). The unique developmental program of the acoel flatworm, *Neochildia fusca*. Dev Biol.

[B60] Sasai Y (2001). Roles of Sox factors in neural determination: conserved signaling in evolution?. Int J Dev Biol.

[B61] Hejnol A, Martindale MQ (2008). Acoel development supports a simple planula-like urbilaterian. Philos Trans R Soc Lond B Biol Sci.

[B62] Finnerty JR, Paulson D, Burton P, Pang K, Martindale MQ (2003). Early evolution of a homeobox gene: the parahox gene *Gsx *in the Cnidaria and the Bilateria. Evol Dev.

[B63] Martinelli C, Spring J (2004). Expression pattern of the homeobox gene *Not *in the basal metazoan *Trichoplax adhaerens*. Gene Expr Patterns.

[B64] Matus DQ, Halanych KM, Martindale MQ (2007). The Hox gene complement of a pelagic chaetognath, *Flaccisagitta enflata*. Integ Comp Biol.

[B65] Hui JH, Holland PW, Ferrier DE (2008). Do cnidarians have a ParaHox cluster? Analysis of synteny around a *Nematostella *homeobox gene cluster. Evol Dev.

[B66] Aronowitcz J, Lowe CJ (2006). *Hox *gene expression in the hemichordate *Saccoglossus kowalevskii *and the evolution of deuterostome nervous system. Integ Comp Biol.

[B67] Wada H, Garcia-Fernandez J, Holland PW (1999). Colinear and segmental expression of amphioxus Hox genes. Dev Biol.

[B68] Hinman VF, O'Brien EK, Richards GS, Degnan BM (2003). Expression of anterior Hox genes during larval development of the gastropod *Haliotis asinina*. Evol Dev.

[B69] Irvine SQ, Martindale MQ (2000). Expression patterns of anterior Hox genes in the polychaete *Chaetopterus*: correlation with morphological boundaries. Dev Biol.

[B70] Kulakova M, Bakalenko N, Novikova E, Cook CE, Eliseeva E, Steinmetz PR, Kostyuchenko RP, Dondua A, Arendt D, Akam M (2007). Hox gene expression in larval development of the polychaetes *Nereis virens *and *Platynereis dumerilii *(Annelida, Lophotrochozoa). Dev Genes Evol.

[B71] Kawakami K, Sato S, Ozaki H, Ikeda K (2000). *Six *family genes--structure and function as transcription factors and their roles in development. Bioessays.

[B72] Ladurner P, Rieger R (2000). Embryonic muscle development of *Convoluta pulchra *(Turbellaria-Acoelomorpha, Platyhelminthes). Developmental Biology.

[B73] Ferrier DEK, Akam M (1996). Organization of the Hox gene cluster in the grasshopper, *Schistocerca gregaria*. Proc Natl Acad Sci USA.

[B74] Bender W, Akam M, Karch F, Beachy PA, Peifer M, Spierer P, Lewis EB, Hogness DS (1983). Molecular Genetics of the Bithorax Complex in *Drosophila melanogaster*. Science.

[B75] Aboobaker AA, Blaxter ML (2003). Hox Gene Loss during Dynamic Evolution of the Nematode Cluster. Curr Biol.

[B76] Ikuta T, Yoshida N, Satoh N, Saiga H (2004). *Ciona intestinalis *Hox gene cluster: Its dispersed structure and residual colinear expression in development. Proc Natl Acad Sci USA.

[B77] Seo HC, Edvardsen RB, Maeland AD, Bjordal M, Jensen MF, Hansen A, Flaat M, Weissenbach J, Lehrach H, Wincker P (2004). Hox cluster disintegration with persistent anteroposterior order of expression in *Oikopleura dioica*. Nature.

[B78] Cameron RA, Rowen L, Nesbitt R, Bloom S, Rast JP, Berney K, Arenas-Mena C, Martinez P, Lucas S, Richardson PM (2006). Unusual gene order and organization of the sea urchin hox cluster. J Exp Zoolog B Mol Dev Evol.

[B79] Boyer BC (1971). Regulative development in a spiralian embryo as shown by cell deletion experiments on the Acoel, *Childia*. J Exp Zool.

[B80] Duboule D (1994). Temporal colinearity and the phylotypic progression: a bias for the stability of a vertebrate Bauplan and the evolution of morphologies through heterochrony. Development, Suppl.

[B81] Ferrier DE, Holland PW (2002). *Ciona intestinalis *ParaHox genes: evolution of Hox/ParaHox cluster integrity, developmental mode, and temporal colinearity. Mol Phylogenet Evol.

[B82] Soshnikova N, Duboule D (2009). Epigenetic temporal control of mouse Hox genes in vivo. Science.

[B83] Tarchini B, Duboule D (2006). Control of Hoxd genes' collinearity during early limb development. Dev Cell.

[B84] Tschopp P, Tarchini B, Spitz F, Zakany J, Duboule D (2009). Uncoupling time and space in the collinear regulation of Hox genes. PLoS Genet.

[B85] Deschamps J (2007). Ancestral and recently recruited global control of the *Hox *genes in development. Curr Opin Genet Dev.

[B86] Moreno E, Nadal M, Baguñà J, Martínez P (2009). Tracking the origins of the bilaterian Hox patterning system: Insights from the acoel flatworm *Symsagittifera roscoffensis*. Evol Dev.

[B87] Guth SI, Wegner M (2008). Having it both ways: Sox protein function between conservation and innovation. Cell Mol Life Sci.

[B88] Kourakis MJ, Master VA, Lokhorst DK, Nardelli-Haefliger D, Wedeen CJ, Martindale MQ, Shankland M (1997). Conserved anterior boundaries of Hox gene expression in the central nervous system of the leech *Helobdella*. Dev Biol.

[B89] Nardelli-Haefliger D, Shankland M (1992). *Lox2*, a putative leech segment identity gene, is expressed in the same segmental domain in different stem cell lineages. Development.

[B90] Wong VY, Aisemberg GO, Gan WB, Macagno ER (1995). The leech homeobox gene *Lox4 *may determine segmental differentiation of identified neurons. J Neurosci.

[B91] Wysocka-Diller JW, Aisemberg GO, Baumgarten M, Levine M, Macagno ER (1989). Characterization of a homologue of bithorax-complex genes in the leech *Hirudo medicinalis*. Nature.

[B92] Salser SJ, Loer CM, Kenyon C (1993). Multiple HOM-C gene interactions specify cell fates in the nematode central nervous system. Genes Dev.

[B93] Diederich RJ, Merrill VK, Pultz MA, Kaufman TC (1989). Isolation, structure, and expression of *labial*, a homeotic gene of the Antennapedia Complex involved in *Drosophila *head development. Genes Dev.

[B94] Krumlauf R, Marshall H, Studer M, Nonchev S, Sham MH, Lumsden A (1993). Hox homeobox genes and regionalisation of the nervous system. J Neurobiol.

[B95] Lumsden A, Krumlauf R (1996). Patterning the vertebrate neuraxis. Science.

[B96] Lee PN, Callaerts P, De Couet HG, Martindale MQ (2003). Cephalopod Hox genes and the origin of morphological novelties. Nature.

[B97] Gionti M, Ristoratore F, Di Gregorio A, Aniello F, Branno M, Di Lauro R (1998). *Cihox5*, a new *Ciona intestinalis *Hox-related gene, is involved in regionalization of the spinal cord. Dev Genes Evol.

[B98] Keys DN, Lee BI, Di Gregorio A, Harafuji N, Detter JC, Wang M, Kahsai O, Ahn S, Zhang C, Doyle SA (2005). A saturation screen for cis-acting regulatory DNA in the Hox genes of *Ciona intestinalis*. Proc Natl Acad Sci USA.

[B99] Keynes R, Krumlauf R (1994). *Hox *genes and regionalization of the nervous system. Annu Rev Neurosci.

[B100] Deutsch J, Le Guyader H (1998). The neuronal zootype. An hypothesis. C R Acad Sci III.

[B101] Hirth F, Kammermeier L, Frei E, Walldorf U, Noll M, Reichert H (2003). An urbilaterian origin of the tripartite brain: developmental genetic insights from *Drosophila*. Development.

[B102] Lichtneckert R, Reichert H (2005). Insights into the urbilaterian brain: conserved genetic patterning mechanisms in insect and vertebrate brain development. Heredity.

[B103] Lowe CJ, Wu M, Salic A, Evans L, Lander E, Stange-Thomann N, Gruber CE, Gerhart J, Kirschner M (2003). Anteroposterior patterning in hemichordates and the origins of the chordate nervous system. Cell.

[B104] Iimura T, Pourquie O (2007). Hox genes in time and space during vertebrate body formation. Dev Growth Differ.

[B105] Fröbius AC, Seaver EC (2006). ParaHox gene expression in the polychaete annelid *Capitella *sp. I. Dev Genes Evol.

[B106] de Rosa R, Prud'homme B, Balavoine G (2005). *Caudal *and *even-skipped *in the annelid *Platynereis dumerilii *and the ancestry of posterior growth. Evol Dev.

[B107] Kulakova MA, Cook CE, Andreeva TF (2008). *ParaHox *gene expression in larval and postlarval development of the polychaete *Nereis virens *(Annelida, Lophotrochozoa). BMC Dev Biol.

[B108] Hinman VF, Becker E, Degnan BM (2000). Neuroectodermal and endodermal expression of the ascidian *Cdx *gene is separated by metamorphosis. Dev Genes Evol.

[B109] Holland PW (2001). Beyond the Hox: how widespread is homeobox gene clustering?. J Anat.

[B110] Wu LH, Lengyel JA (1998). Role of *caudal *in hindgut specification and gastrulation suggests homology between *Drosophila *amnioproctodeal invagination and vertebrate blastopore. Development.

[B111] Hejnol A, Martindale MQ, Telford MJ, Littlewood DTJ (2009). The mouth, the anus and the blastopore - open questions about questionable openings. Animal Evolution: genes, genomes, fossils and trees.

[B112] Shannon T, Achatz JG (2007). *Convolutriloba macropyga *sp. nov., an uncommonly fecund acoel (Acoelomorpha) discovered in tropical aquaria. Zootaxa.

[B113] Guindon S, Gascuel O (2003). A simple, fast, and accurate algorithm to estimate large phylogenies by maximum likelihood. Syst Biol.

[B114] Edgar RC (2004). MUSCLE: multiple sequence alignment with high accuracy and high throughput. Nucleic Acids Res.

[B115] Banerjee-Basu S, Baxevanis AD (2001). Molecular evolution of the homeodomain family of transcription factors. Nucleic Acids Res.

[B116] Jekely G, Arendt D (2007). Cellular resolution expression profiling using confocal detection of NBT/BCIP precipitate by reflection microscopy. Biotechniques.

[B117] Hessling R (2003). Novel aspects of the nervous system of *Bonellia viridis *(Echiura) revealed by the combination of immunohistochemistry, confocal laser scanning microscopy and three-dimensional reconstruction. Hydrobiologia.

