# Supplementary material for: Coordinated spatial and temporal expression of Hox genes during embryogenesis in the acoel Convolutriloba longifissura
Source: BMC Biol. 2009 Oct 1;7:65. doi: 10.1186/1741-7007-7-65 (PMC2761877; doi:10.1186/1741-7007-7-65)
Supplement: Additional file 1 — Additional figures and table. Additional Data Figure S1 - Phylogenetic analysis of Hox and ParaHox genes that excluded most cnidarian orthologs. Additional Data Figure S2 - Bayesian orthology assignment of ClSoxB1. Additional Data Table S1 - Motif comparisons of acoel Hox genes with that of other bilaterians and cnidarians. [file 1741-7007-7-65-S1.PDF]

Additional Data

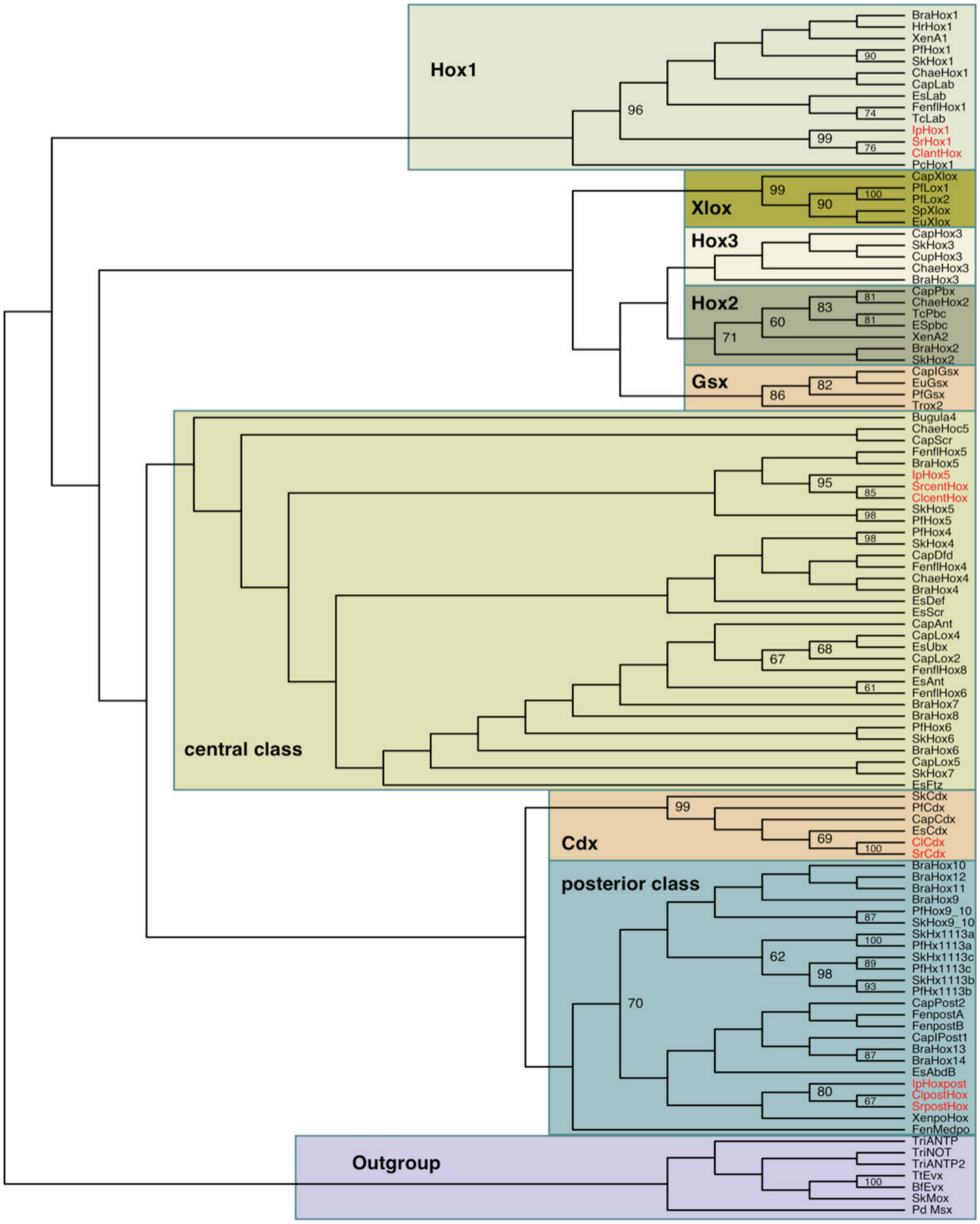

Supplementary Figure 1: Maximum Likelihood Phylogenetic without cnidarian *Hox* orthologs. *Hox* and *ParaHox* genes of acoels indicated in red. Bootstraps (3000) above 60 are indicated in percentage.

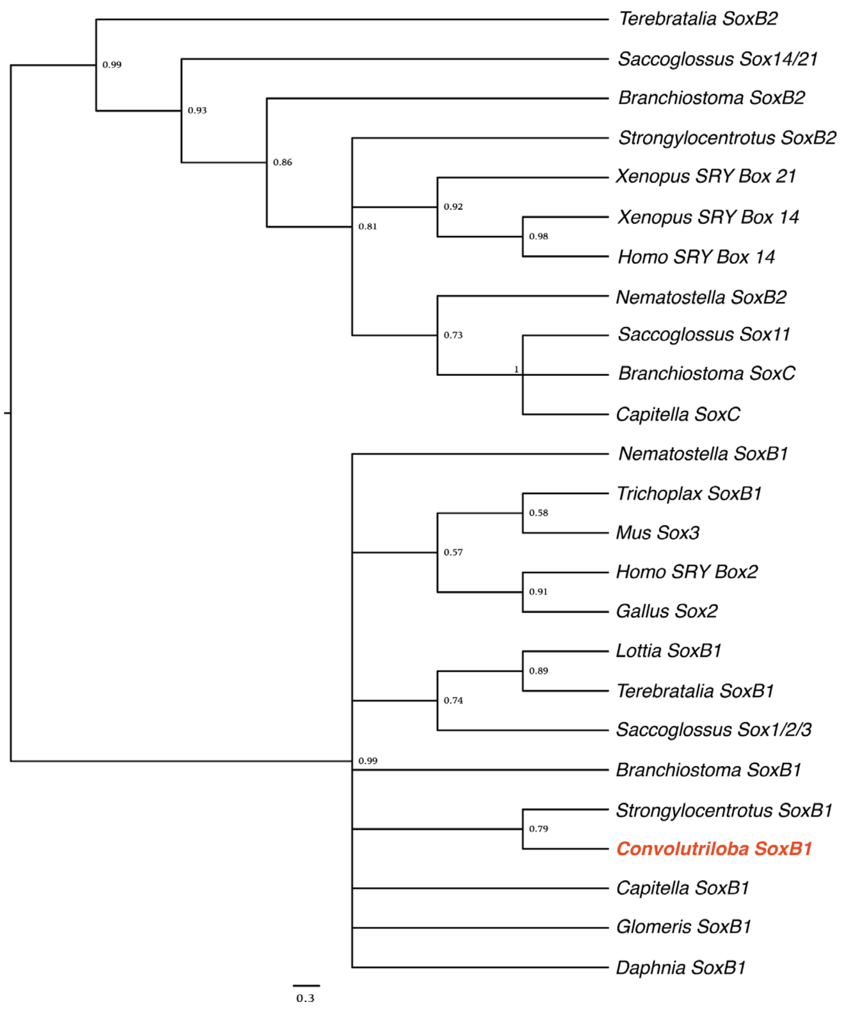

Supplementary Figure 2: Bayesian phylogenetic orthology assignment of the *ClSoxB1.*

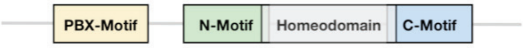

Hox/paraHox Class	Species	PBX-Motif	presence	distance from Homeodomain	N-Motif	N-Name	C-Motif	C-Name
**Hox1**	*C. longifissura*	QFAWMK	✔	38	PGVQTRGGRTNFTNK	Na	KLLKEGKLS--	-
	I. pulchra	--SWMK	✔	28	PGSQTRGGRTNFTNK	Na	KLVKEGKLA--	-
	S. roscoffensis	PFSWMK	✔	37	PGVQTRGGRTNFTNK	Na	KLLKEGKLS--	-
	S. kowalewski	MYNWMK	✔	17	FTGSPANGRTNFTNK	Na	KQKKRFKDGPS	-
	Capitella lab	TYKWMT	✔	21	FAGQPNMGRTNFTNK	Na	KRLKENTSTTP	-
**Hox2**	*S. kowalewski*	MYNWMK	✔	20	EAGHHRRVRTAFTNT	Nb	RQIMKAAVSGS	-
	Capitella pb	EYPWMR	✔	55	ASNHPRRLRTAYTNT	Nb	RQTGKGSGNSP	-
**Hox3**	*S. kowalewski Hox3*	MYAWMT	✔	25	GRSPIKRMRTNFCTA	Nb	KDKGLVDPYQA	-
	Capitella Hox3	IYPWMR	✔	40	VGQPSKRARTAYTSA	Nb	KDQKQKNLMEK	-
**central Hox**	*C. longifissura*	MFAWMS	✔	3	PPTDCKRTRTAYTRF	Nb	KDNNLKSMSQV	Ca
	I. pulchra	?	?	?	?	?	KDNNLKSMSQV	Ca
	S. roscoffensis	VFAWMS	✔	3	PPTDCKRTRTAYTRF	Nb	KDNNLKSMSQI	Ca
	S. kowalewski Hox4	MYPWMK	✔	15	NGAEHKRTRTAYTRY	Nb	KDHNLPNTKTK	Ca
	S. kowalewski Hox5	VYPWMR	✔	10	NGMEAKRSRTAYTRY	Nb	KEHNVKSISQI	Ca1
	Capitella dfd	IYPWMK	✔	14	FVGDSKRTRTAYTRH	Nb	KEHKLPNTKTR	Ca
	Capitella scr	IYPWMK	✔	7	SNADNKRTRTSYTRH	Nb	KEHKLAHLAKS	Ca1
	S. kowalewski Hox6	IYPWMR	✔	5	CGSDQRRGRQTYTRY	Nb	KEQKTGGAPSP	Ca
	S. kowalewski Hox7	LYPWVN	✔	5	PEVPKKRCRQTYTRY	Nb	KESKKDDGENS	Ca
	Capitella lox5	IYPWMR	✔	8	FGYEQKRTRQTYTRY	Nb	KENNISKLTGP	Ca
	Capitella antp	?	-	?	AGPERKRGRQTYTRY	Nb	KENRQIEVLRQ	Ca
**posterior Hox**	*C. longifissura*	SPGWLV	reduced	3	VRNVSRKKRRPYTKN	Nbx	KQHSGGPGLGP	-
	I. pulchra	-MGWIA	reduced	3	ARNVSRKKRRPYTKT	Nbx	KQMNGGTPQTM	-
	S. roscoffensis	GMAWMA	✔	3	ARNVSRKKRRPYTKN	Nbx	KQHSGGPGMGP	-
	Branchiostoma Hox9	QPGWMN	✔	3	NNHSSRKKRCPYTRF	Nbx	KMSKQRQEQQQ	-
	Ptychodera 9/10	PPNWMT	✔	3	TSASGRKKRCPYTKF	Nbx	KQNHRNTICMI	-
	Strongylocentrotus 11/13	TPSWMF	✔	3	AALPRRTKRRPYTKL	Nbx	KQKKMNEREKR	-
	Capitella Post1	------	-	-	SDVNPKKKRKPYSKP	Nbx	KEKKIATKRAK	-
	Capitella Post2	------	-	-	DAPKQRKKRKPYTRY	Nbx	KRKKLNERAKS	-
**Gsx**	*Capitella*	------	-	-	SSDAVKRMRTAFSST	Nb	KEGAAHGSRDH	-
	Ptychodera	------	-	-	NLQSSKRIRTAFTST	Nb	KRRKTRVRDCR	-
**Xlox**	*Capitella*	HFPWMK	✔	21	TFSENKRTRTAYTRA	Nb	KDVDKKRPQQS	-
	Strongylocentrotus	QFPWMK	✔	21	DFDENKRTRTAYTRA	Nb	KEEAKRKPLKQ	-
**Cdx**	*C. longifissura*	------	-	-	KTRTKDKYRVVYTDR	-	KVSRKVPGGGN	-
	S. roscoffensis	------	-	-	KTRTKDKYRVVYTDR	-	KVSRKVPGGGN	-
	S. kowalewski	PYAWMR	✔	13	KTRTKDKYRVVYTDH	-	KERKQNKKKVM	-
	Capitella	PYDWMK	✔	14	KTRTKDKYRIVYSEY	-	KQKRKMEEALS	-
**Nv AntHox1**	postHox	------	-	-	KQPFKHRKRMAYTRI	-	KDNKPVIPTSR	-
**Nv AntHox1a**	postHox	DAPWHY	✔	3	KQPFKHRKRMAYTRI	-	KDNKPVIPTSR	-
**Nv AntHox2**	gsx?	------	-	-	GNSRSKRIRTAYTSM	Nb	KDKKAAQHGTT	-
**Nv AntHox7**	hox2	IYPWMR	✔	11	GGKHTKRYRTSYTNR	Nb	KDEKQKEDGPS	-
**Nv Anthox6**	hox1	------	-	-	NPSPSQKKRFTFTQR		REFKDSLQKPA	-
**Nv AntHox6a**	(HDV060)	IYPWMT	✔	14	ECTSDKNRTIYSTRQ		KENKLVEKAIE	-
**Nv AntHox8**	hox2?	IYPWMK	✔	12	FVRESKRHRTSYTNK			
**Nv AntHox8b**	Hox2?	IYPWMK	✔	12	FVRESKRHRTSYTNK		KDEKQKTEDCY	-
**Nv HD065**	(Cdx/Xlox)	------		-	SAQVRSRARTAYTAS		KTDSNGAFEGV	
**Nv Anthox9**	(HDV117)	VYPWMV	✔	21	SSTSNHAPRSAFTTV		REAKNVEEVLH	-
**Che9-14A**	pHox?	YFPWNC	✔	4	CFTDGKRKRTSYTRR		REKPTSTKNEN	-
**Che9-14B**	pHox?	------	-	-	DSNGHRRKRTAYSRA			
**Che9-14C**	pHox?	AGPWIC	✔	4	RDIDTKRKRMTYSRK		KECNKKKTGAV	-
**CheHox1**	pHox?	NYQWMN	✔	59	ATGEQGKKRICFSQK	-	REMKGKRFQLR	-
**CheCdx**	cdx?	------	-	-	SQDPTMRSRPCFSSH	-	KQKKISGEVIE	-
**CheGsx**	gsx?	------	-	-	GGSKSKRIRTAYTSI	Nb	KDKKSGFNNSY	
**TurrPdx**	xlox?	------	-	-	SAKVKKRNRTTYTRV	-	RDEEDALRNEN	-

Supplementary Table 1: Motif comparison of the acoel *Hox* genes with that of bilaterian and cnidarian sequences.
